# Choosing preferable labels for the Japanese translation of the Human Phenotype Ontology

**DOI:** 10.5808/GI.2020.18.2.e23

**Published:** 2020-06-18

**Authors:** Kota Ninomiya, Terue Takatsuki, Tatsuya Kushida, Yasunori Yamamoto, Soichi Ogishima

**Affiliations:** 1 National Institute of Public Health, Wako 351-0197, Japan; 2 Social Cooperation Program of IT Healthcare, Graduate School of Pharmaceutical Sciences, The University of Tokyo, Tokyo 113-0033, Japan; 3 Database Center for Life Science, Research Organization of Information and Systems, Kashiwa 277-0871, Japan; 4 BioResource Research Center, RIKEN, Tsukuba 305-0074, Japan; 5 National Bioscience Database Center, Japan Science and Technology Agency, Tokyo 102-8666, Japan; 6 Advanced Research Center for Innovations in Next-Generation Medicine, Tohoku University, Sendai 980-8573, Japan

**Keywords:** biological ontologies, natural language processing, phenotype, rare diseases, translations

## Abstract

The Human Phenotype Ontology (HPO) is the de facto standard ontology to describe human phenotypes in detail, and it is actively used, particularly in the field of rare disease diagnoses. For clinicians who are not fluent in English, the HPO has been translated into many languages, and there have been four initiatives to develop Japanese translations. At the Biomedical Linked Annotation Hackathon 6 (BLAH6), a rule-based approach was attempted to determine the preferable Japanese translation for each HPO term among the candidates developed by the four approaches. The relationship between the HPO and Mammalian Phenotype translations was also investigated, with the eventual goal of harmonizing the two translations to facilitate phenotype-based comparisons of species in Japanese through cross-species phenotype matching. In order to deal with the increase in the number of HPO terms and the need for manual curation, it would be useful to have a dictionary containing word-by-word correspondences and fixed translation phrases for English word order. These considerations seem applicable to HPO localization into other languages.

**Availability:** As these new translations are still in progress, only an old version of Japanese translations can be obtained from https://github.com/ogishima/HPO-japanese.

## Introduction

The Human Phenotype Ontology (HPO) [1] is the de facto standard ontology to describe human phenotypes. Increasingly many researchers have been using it for accurate phenotype-driven diagnoses and translational research. As the HPO has been used by an increasingly diverse group of researchers since it was first released in 2008, its content has continued to expand. This new content includes the translation of HPO terms from English into several languages [2]. As translation into Japanese has been conducted since the BioHackathon 15 (BH15) [3], there are several Japanese translations for each English HPO term. At the Biomedical Linked Annotation Hackathon 6 (BLAH6), an attempt was made to select preferable unique Japanese terms.

The HPO has mainly been used in the field of rare diseases as the most comprehensive resource for deep phenotyping, which is defined as “the precise and comprehensive analysis of phenotypic abnormalities in which the individual components of the phenotype are observed and described” [4]. As approximately 80% of rare diseases, the number of which is estimated to be between 5,000 and 8,000, are thought to be genetic [5,6], they may occur anywhere.

For individuals with rare diseases, delays in diagnoses and frequent misdiagnoses lead to irreversible disease progression, and mistreatment based on a misdiagnosis can even harm patients in some circumstances. This problematic journey faced by patients with rare diseases is sometimes called the “diagnostic odyssey.” It has reported that it takes 5–7 years on average for patients with rare diseases to be diagnosed correctly in the UK and USA, and that patients received incorrect diagnoses two or three times [7]. In Japan, the average time to be diagnosed correctly with Fabry disease was also found to be about 20 years [8].

Therefore, HPO localization is expected to help clinicians who are not fluent in English make early diagnoses based on medical records containing standardized and detailed phenotypic information. HPO terms are being translated into Japanese, French, German, Russian, Turkish, Spanish, Italian, Dutch, Portuguese, and Chinese.

In order to understand the pathology of a specific disease, researchers often use model animals that present the same symptoms or have the same genetic abnormalities. When they choose the appropriate model animals, standardized phenotyping can be a critical clue. In Exomiser [9], phenotypic data from several species, such as mice and zebrafish, are also used for functional annotation of genetic variants from human whole-genome sequencing data. The standardized description of phenotypes by the HPO and other phenotype ontologies has enabled a phenotype-based comparison of species through cross-species phenotype matching. Harmonization of translations is also expected to make it possible for researchers to search for bio-resources for human beings or other species only using the same terms in Japanese.

At BH15, which was held in 2015, HPO terms started to be translated into Japanese. As a result of the hackathon and subsequent efforts, each HPO term had four Japanese equivalent terms, which were translated using different English-Japanese dictionaries, and the translations have been made available to the public [10].

One of the four translations is based on the Life Science Dictionary (LSD) [11], which is an English-Japanese dictionary for the life sciences; this translation is updated by researchers at Kyoto University. The second translation is based on the Japanese translation of the Mammalian Phenotype (MP) ontology [12], and was created by Riken BioResource Research Center. The third translation was created by Kenji Naritomi, a medical expert who has translated many materials about genetic diseases into Japanese. He translated the HPO terms to the extent that he could. The last translation is an automatic translation using Google Translate.

At BLAH6, a unique Japanese translation for each English term in the four translations was selected through trial and error based on the criterion that translated terms should not sound anomalous or unnatural in Japanese. Translations were prepared for the 10,668 HPO terms as of October 2017.

As the HPO describes the phenotypes of human beings and the MP describes those of mammals, they have many concepts in common. The equivalence of their concepts has already been explored by Mungall [13]. At BLAH6, the relationship between the Japanese translation of the HPO and that of the MP was examined with the goal of harmonizing them so that researchers could easily search for biological resources, using the same expression for the same phenotype. In this comparison, the Japanese translations made by Kenji Naritomi were adopted as the counterparts of the MP Japanese terms.

## Methods

First, a rule-based method was used to choose the most appropriate translated terms in the following order.

1. If two or more translated terms were the same among the four translations, they were chosen as a unique Japanese term. If there were two sets of words, such that two of the four translations were the same, and the other two were the same, they were labeled as “two appropriate candidates determined by a majority.” The rest of these cases were labeled as "a unique Japanese term" and "determined by a majority."

2. For the rest of the HPO terms, a morphological analysis was conducted using Mecab [14], with the MANBYO dictionary [15] as a user dictionary, for all Japanese translation candidates except those based on the LSD. Then, candidates for preferable labels were automatically chosen based on whether the morphological analysis indicated that the terms included anomalous features, defined as below. The MANBYO dictionary contains a large number of medical terms in Japanese. As some of the terms derived from the LSD are combinations of translated words, they were excluded from this analysis.

As no consensus necessarily exists regarding the precise definition of “anomalous” features, the terms were separately labeled with each feature to make it possible to change the criteria used to identify anomalous terms. The features of anomalous terms were as follows:

(1) Terms including verbs or ending with a non-noun word (e.g., 出生時にみられ時間とともに真っすぐなる大腿骨湾曲). These features seem anomalous because HPO terms are supposed to be nouns, and it is preferable for combinations to only involve nouns.

(2) Terms including particles or adjectival verbs (e.g., 尺骨の有力な茎状突起), for the same reason as (1).

(3) Terms including adjectives (e.g., 幅広い長管骨), also for the same reason as (1).

(4) Terms including Japanese commas, which appear much more unusual than English commas when they are used in terms (e.g., 異所性心臓、心臓転位).

(5) Terms including untranslated English words (e.g., 角膜 stromal 浮腫).

(6) Cases where English terms were not translated at all for unknown reasons, and the translated terms were blank.

3. In this analysis, all the anomalous features were adopted. Candidate terms were ranked in the following order.

(1) If a translated term included some strange features, it was excluded from consideration.

(2) If only one term was left after the exclusion of anomalous terms, it was chosen as the most appropriate one. Such terms were labeled as “a unique Japanese term” and “determined by an exclusion process.”

(3) If more than two terms were left, it was difficult to choose which was better, and such cases were labeled as “multiple appropriate candidates determined by an exclusion process.”

(4) If all of the terms were excluded, the item was labeled as “no appropriate candidates determined by an exclusion process.” If all the translated terms were initially blank, they were labeled as "BLANK."

Second, an attempt was made to find out how equivalent concepts between HPO and MP are described in English and Japanese to promote the consistency of translations between these resources. As the equivalence data only contain the IDs of concepts, the English and Japanese terms were collected using the Japanese translation of the HPO [10], with HPO data as of August 2015 and July 2016, and the relationship between HPO and MP was assessed based on the MP data as of October 2012.

## Results

The results of labeling all the HPO terms are shown below (Table 1).

**Fig. 1.**
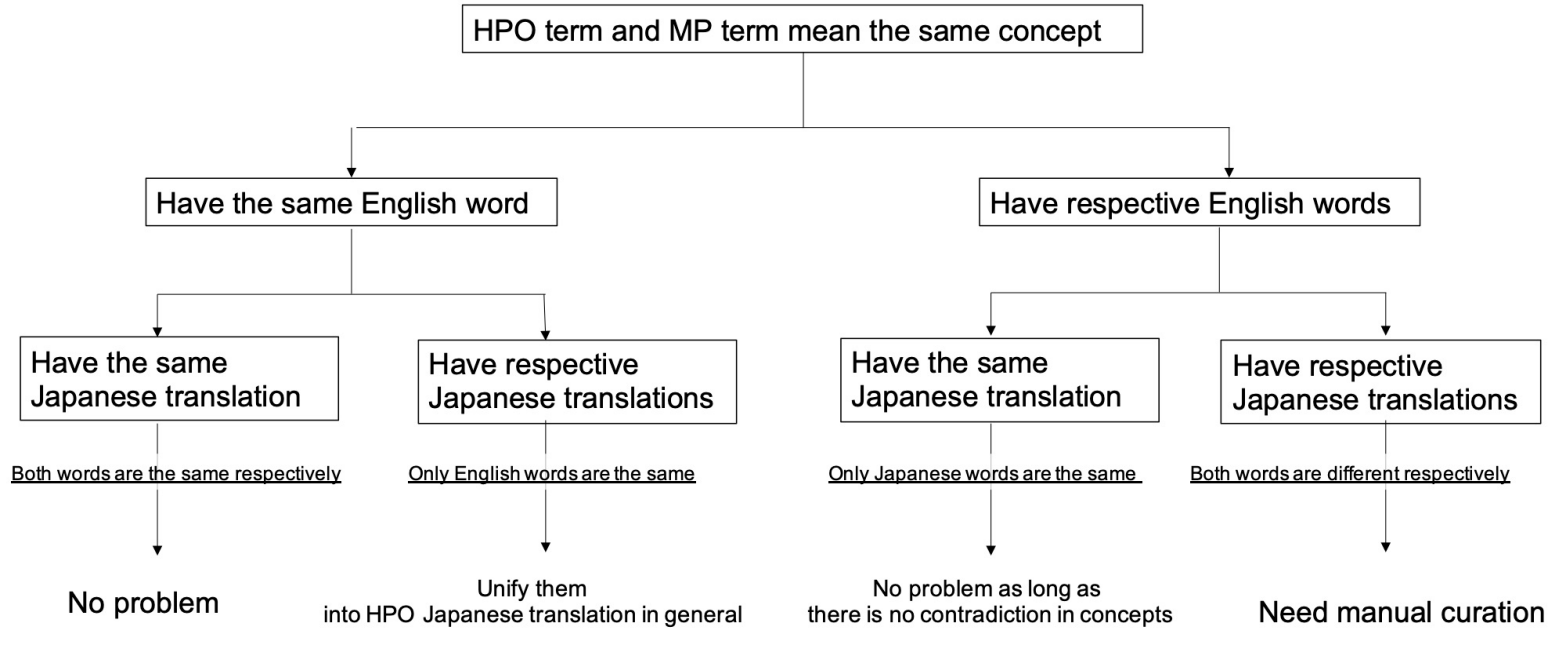
Analysis of the relationships between the Human Phenotype Ontology (HPO) and Mammalian Phenotype (MP) and suggestions regarding how to harmonize these resources.

In the second phase, the relationships between HPO and MP concepts in Japanese and English were explored, and ways to harmonize the translations were examined. A flow chart is shown below (Fig. 1).

All the HPO and MP terms referring to the same concepts were divided into the four categories described in the flow chart. As the equivalence data were created after the first translation attempt in 2015, some terms had no Japanese translation candidates. The results of a character-string comparison between them are as follows (Table 2).

## Discussion

In this trial, about half of the HPO terms were found to have a unique Japanese translation. However, there are three points to consider regarding these labels.

First, those labeled as “determined by a majority” sometimes included anomalous Japanese expressions, as terms were not excluded based on anomalous features if they were identical in a majority of sources. Therefore, the order of assigning labels should perhaps be reconsidered.

Second, HPO terms that had two appropriate candidates determined by a majority were divided into three groups, although they had the same problem as those labeled as “determined by a majority.” The first group included terms with only slight differences, such as whether or not they included “症”, which means “syndrome” in Japanese (e.g., 不眠|不眠症). Therefore, such typical and almost meaningless characters or words should be omitted as stop words in the next matching trial. The second group contained translations that had entirely different meanings (e.g., 硬化症|第1中足骨硬化症). In this case, one of the options must be a mistranslation. A possible reason for this is that some words in the terms were ignored in translation because the translation systems did not contain them in their dictionaries and could not recognize them properly. The last group required manual curation because the order and the selection of translated words were different (e.g., 髄様甲状腺癌|甲状腺髄様癌).

Finally, problems in Japanese translation labeling related to the exclusion process are mainly caused by the definition of anomalous features and the accuracy of the morphological analysis. Therefore, the definitions need to be made more sophisticated in future trials by adding or removing exclusion criteria. It is also important to choose an appropriate morphological analyzer for dealing with medical expressions, such as Juman++ [16] or Sudachi [17].

The relationship between the HPO and MP translations was classified into four categories according to character-string comparisons. First, if the English and Japanese terms are both the same, there is nothing to change. Second, if only the English terms are the same, the HPO translations take precedence over the MP translations, and the latter is unified to follow the former, as the former already seems to be used for more diverse purposes and to be more widespread. There are two reasons for inconsistencies in Japanese translations. One is the same as encountered for Japanese localized terms assigned the label “two appropriate candidates determined by a majority.” The other is that the same terms, especially those that refer to morphological abnormalities of external body parts (instead of abnormal internal situations), are sometimes translated differently depending on the species. For example, the words “male” and “female” are “男性” and “女性” for human beings, respectively. However, for non-human mammals such as mice and rats, these terms are written as “オス” and “メス”, respectively. Therefore, the principle of assigning precedence to the HPO translations is acceptable only in a general sense. Third, if only the Japanese terms are the same, there is no need to change the translation as long as the concepts are similar between the HPO and MP terms. Finally, if both the English and Japanese terms are different, there is no option other than manual curation. Since applying these principles led to the finding that roughly half of the terms need manual curation to be harmonized, another way needs to be found to decrease the necessity for manual curation in further research.

As the HPO includes technical terms, orthodox translations that are generally accepted among health professionals should be adopted. An excellent approach would seem to be to map these terms to other dictionaries for translation and to adapt their translations if doing so is permissible because dictionaries are thought to be edited according to the same policy. This approach seems to contribute to external consistency among dictionaries and to reinforce the stability of orthodox translations. Nonetheless, the MP translations can be candidates for replacing the HPO translations, as they sometimes contains better expressions, and a comparison between them enables harmonization and cross-species matching or searching. If translations of the terms cannot be found in other resources, or there are several translation candidates, experts need to translate them manually. Although this task requires extensive work and costs, it is ultimately unavoidable.

To deal with the increase of the number of HPO terms and the excessive dependence on manual curation—despite its inevitability in principle—it may be a good idea to develop a dictionary that contains word-by-word correspondences based on the temporarily completed translations of the HPO and MP. Such a dictionary would enable the generation of translation candidates for new terms consistent with the fixed HPO and MP translations created previously. As some word orders are common in English terms, it is possible to establish fixed Japanese phrases for each of these frequent word orders. Therefore, dictionaries and lists of fixed phrases can reduce the task of manual curation by changing it from translation of terms from scratch to only selection of the most appropriate candidates. These approaches seem to be applicable to HPO localization into other languages.

## Conclusion

In this study, an attempt was made to determine a single unique translation for each term in the HPO in a rule-based way. For about half of the terms, only one appropriate Japanese word was identified, and for the rest, manual curation was needed. However, as this approach yielded insufficient accuracy, further consideration is necessary and will be given in venues such as another future hackathon.

The relationship between the HPO and MP was also investigated to evaluate the task of establishing consistency between them. Based on the analysis, the translations of both ontologies should be harmonized to improve their usability for annotating phenotypes of humans and non-human mammals.

It is possible that the number of HPO terms will continue to increase and that there will be more need for manual curation. An effective approach would seem to be to create a dictionary that contains word-by-word correspondences based on the temporary translations and fixed translation phrases for English terms in word orders that frequently appear. These approaches are most likely applicable to HPO localization into other languages.

## Figures and Tables

**Table 1.** Summary table of the labels assigned to all the HPO terms

Label	No.
All the HPO terms	10,668
A unique Japanese term	5,678
Determined by a majority	3,096
Determined by an exclusion process	2,687
Two appropriate candidates determined by a majority	105
Multiple appropriate candidates determined by an exclusion process	2,165
No appropriate candidates determined by an exclusion process	2,720
BLANK	5

HPO, Human Phenotype Ontology.

**Table 2.** Summary table of the categories assigned to HPO/MP terms with the same concepts

Category	No.
All pairs of HPO/MP terms with the same concepts	1,442
Both words are the same in both languages	219
Only the English words are the same	420
Only the Japanese words are the same	115
Both words are different in both languages	688
Japanese translation candidates do not exist yet	128

HPO, Human Phenotype Ontology; MP, Mammalian Phenotype.
